# Platelet-Released Growth Factors and Platelet-Rich Fibrin Induce Expression of Factors Involved in Extracellular Matrix Organization in Human Keratinocytes

**DOI:** 10.3390/ijms21124404

**Published:** 2020-06-20

**Authors:** Andreas Bayer, Bernard Wijaya, Lena Möbus, Franziska Rademacher, Meno Rodewald, Mersedeh Tohidnezhad, Thomas Pufe, Daniel Drücke, Regine Gläser, Jürgen Harder

**Affiliations:** 1 Institute of Anatomy, Kiel University, 24098 Kiel, Germany; 2 Department of Dermatology, Kiel University, 24105 Kiel, Germany; wjybernard@gmail.com (B.W.); lmoebus@dermatology.uni-kiel.de (L.M.); frademacher@dermatology.uni-kiel.de (F.R.); stu104302@mail.uni-kiel.de (M.R.); rglaeser@dermatology.uni-kiel.de (R.G.); jharder@dermatology.uni-kiel.de (J.H.); 3 Institute of Anatomy and Cell Biology, RWTH University of Aachen, 52072 Aachen, Germany; mtohidnezhad@ukaachen.de (M.T.); tpufe@ukaachen.de (T.P.); 4 Department of Reconstructive Surgery, University Hospital Schleswig-Holstein, Campus Kiel, 24105 Kiel, Germany; daniel.druecke@uksh.de

**Keywords:** platelet-released growth factors (PRGF), wound healing, extracellular matrix, keratinocytes

## Abstract

Platelet-released growth factor (PRGF) is a thrombocyte concentrate lysate which, like its clinically equivalent variations (e.g., Vivostat PRF^®^ (platelet-rich fibrin)), is known to support the healing of chronic and hard-to-heal wounds. However, studies on the effect of PRGF on keratinocytes remain scarce. This study aims to identify genes in keratinocytes that are significantly influenced by PRGF. Therefore, we performed a whole transcriptome and gene ontology (GO) enrichment analysis of PRGF-stimulated human primary keratinocytes. This revealed an increased expression of genes involved in extracellular matrix (ECM) organization. Real-time polymerase chain reaction (PCR) and enzyme-linked immunosorbent assay (ELISA) analysis confirmed the PRGF-mediated induction of selected ECM-related factors such as transforming growth factor beta-induced protein, fibronectin 1, matrix metalloproteinase-9, transglutaminase 2, fermitin family member 1, collagen type I alpha 1 and collagen type XXII alpha 1. PRGF-induced expression of the above factors was influenced by blockade of the epidermal growth factor receptor (EGFR), a receptor playing a crucial role in wound healing. A differential induction of the investigated factors was also detected in skin explants exposed to PRGF and in experimentally generated in vivo wounds treated with Vivostat PRF^®^. Together, our study indicates that the induction of ECM-related factors may contribute to the beneficial wound-healing effects of PRGF-based formulations.

## 1. Introduction

Chronic or hard-to-heal wounds display a major problem for health care systems worldwide as they have an immense economic and social impact [1,2]. Their therapy is often difficult, lengthy and expensive [3,4,5,6]. The quality of life of patients affected is significantly impaired [7,8]. One emerging option for the treatment of chronic or hard-to-heal wounds is the local application of autologous thrombocyte concentrate lysates as platelet-released growth factors (PRGF) or platelet-rich fibrin (e.g., Vivostat PRF^®^) that are supposed to contain several cytokines and growth factors [9,10,11] stimulating wound healing [12,13]. In general, PRGF is supposed to induce growth and/or differentiation of influenced cells [14,15] and is used in many medical disciplines for tissue regeneration [16,17,18]. Therefore, PRGF-based treatments are a promising approach in many fields of regenerative medicine [19]. In the context of chronic or hard-to-heal wounds PRGF or its clinically used equivalents for example, Vivostat PRF^®^ have been often used successfully to support wound healing [12,20,21]. Still, the underlying mechanism is analyzed insufficiently. Recently, we have shown that PRGF and Vivostat PRF^®^ induced the expression of antimicrobial peptides [22,23,24] and influenced keratinocytes differentiation [25] and proliferation [26]. These data suggest a profound influence of PRGF on keratinocytes physiology.

To get a general overview of PRGF-influenced genes in keratinocytes we performed a whole transcriptome sequence analysis of human primary keratinocytes treated with PRGF. This revealed an induction of various genes involved in extracellular matrix (ECM) organization. Since ECM remodeling is an important process in wound healing we further analyzed the in vitro, ex vivo and in vivo influence of PRGF on the expression of selected factors associated with ECM physiology. Here we demonstrate that PRGF induces various ECM-related genes suggesting a major role of PRGF in modulating ECM.

## 2. Results

### 2.1. Whole Transcriptome Analyses of PRGF-Stimulated Human Keratinocytes Revealed Induction of Factors Involved in Extracellular Matrix Organization

**Figure 1:**
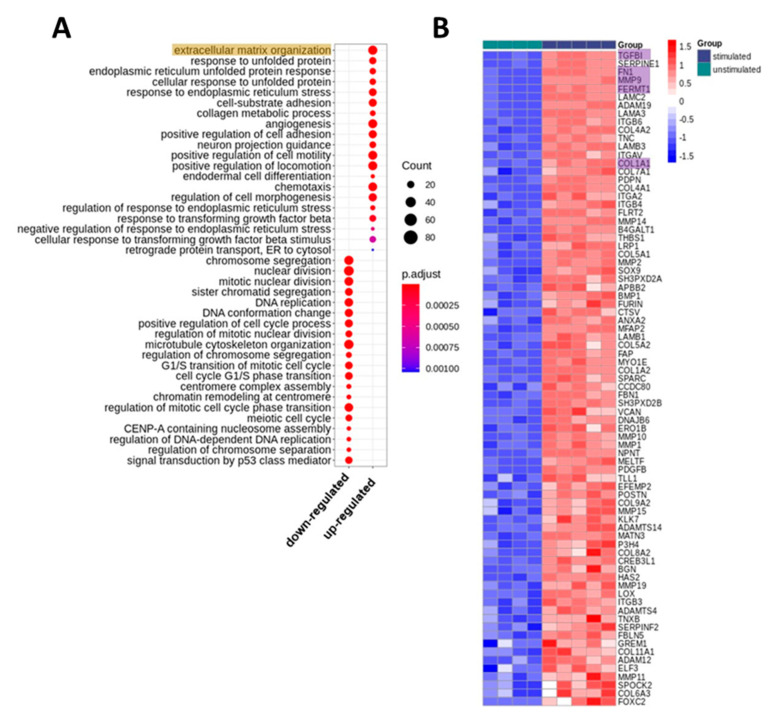
Transcriptomic changes in keratinocytes induced by stimulation with platelet-released growth factor (PRGF). (**A**) Gene Ontology (GO) biological processes that were enriched with genes differentially expressed in PRGF-stimulated vs. unstimulated keratinocytes. The top 20 (according to *p*-value) significantly enriched biological processes for up- and downregulated genes, respectively, are shown. The size of the dots displays the number of differentially expressed genes among the particular biological process. The color displays the adjusted *p*-values. (**B**) Heatmap of genes differentially expressed in stimulated vs. unstimulated keratinocytes from the top enriched GO biological process “extracellular matrix organization.” The color code displays row Z-score. The genes are ordered by log2 fold change and expression level over all samples, that is, the top genes revealed high log2 fold changes together with high expression. The expression of the highlighted genes was further analyzed in detail (see below).

To get an overview of the impact of PRGF on keratinocytes we treated human primary keratinocytes with PRGF derived from 5 different donors and analyzed changes in gene expression by whole transcriptome sequencing (RNA-Seq). We excluded the data of one unstimulated sample from all analysis since principle component analysis showed strong deviation from the other unstimulated samples on transcriptome-wide level. Stimulated keratinocytes revealed 1459 upregulated and 1398 downregulated genes as compared to unstimulated keratinocytes (Supplementary Table S1). Gene ontology (GO) enrichment analysis revealed strong upregulation of factors involved in extracellular matrix (ECM) organization and cell morphology and motility (Figure 1). In contrast, downregulated genes in stimulated keratinocytes were mainly related to DNA replication and cell cycle.

### 2.2. Real-time PCR Confirmed PRGF-Mediated Induction of Selected ECM-Associated Factors in Human Primary Keratinocytes

**Figure 2:**
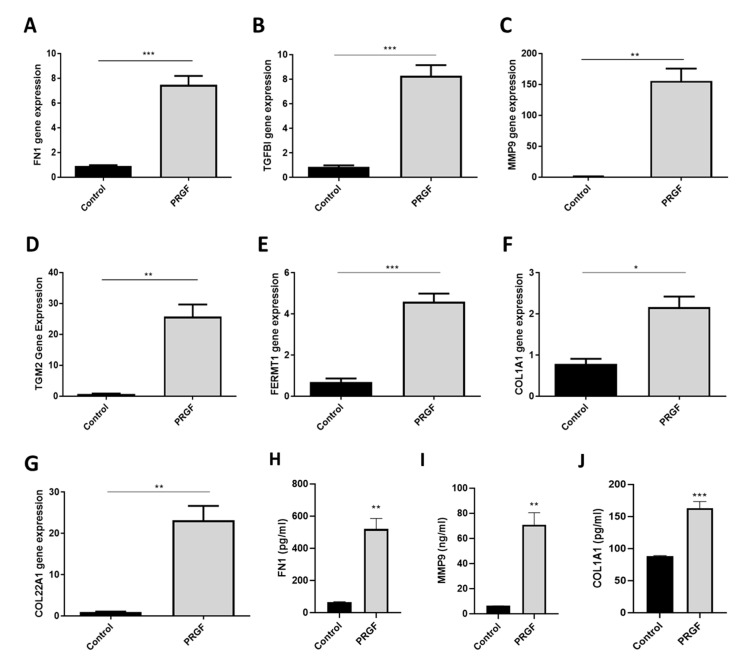
PRGF induces gene expression of various extracellular matrix (ECM)-related proteins in human keratinocytes. Human primary keratinocytes were stimulated for 20 h with PRGF preparations derived from 10 different donors. Relative gene expression of FN1, TGFBI, MMP9, TGM2, FERMT1, COL1A1 and COL22A1 was analyzed by real-time polymerase chain reaction (PCR) (**A**–**G**). Protein release of keratinocytes stimulated for 20 h with PRGF preparations from three different donors was analyzed by enzyme-linked immunosorbent assay (ELISA) specific for FN1, MMP9 and COL1A1 (**H**–**J**). Shown are means ± s.e.m (* *p* < 0.05, ** *p* < 0.01, *** *p* < 0.001; Student’s *t*-test).

The sequencing data and the GO biological process analysis revealed that PRGF induced various genes associated with ECM organization in human primary keratinocytes. Therefore, we further verified the expression of selected candidate genes involved in ECM organization using real-time PCR. The following genes were selected based on abundance and induction level as well as GO classification into ECM organization (see also Figure 1B)—transforming growth factor beta-induced protein (TGFBI), fibronectin 1 (FN1), matrix metalloproteinase-9 (MMP9), transglutaminase 2 (TGM2), fermitin family member 1 (FERMT1) and collagen type I alpha 1 (COL1A1) collagen type XXII alpha1 (COL22A1). According to the whole transcriptome analysis all these genes were abundantly expressed in human primary keratinocytes and strongly upregulated by PRGF treatment. In line with these results, real-time PCR analyses revealed gene induction of all investigated proteins in human primary keratinocytes stimulated with PRGF preparations derived from 10 different donors (Figure 2A–G). To analyze whether gene induction correlates with protein release we also determined protein levels of FN1, MMP9 and COL1A1 in the supernatants of PRGF-stimulated primary keratinocytes. This revealed a significant PRGF-induced release of all investigated proteins (Figure 2H–J).

### 2.3. Time-Dependent Induction of ECM-Related Genes in PRGF-Treated Keratinocytes

**Figure 3:**
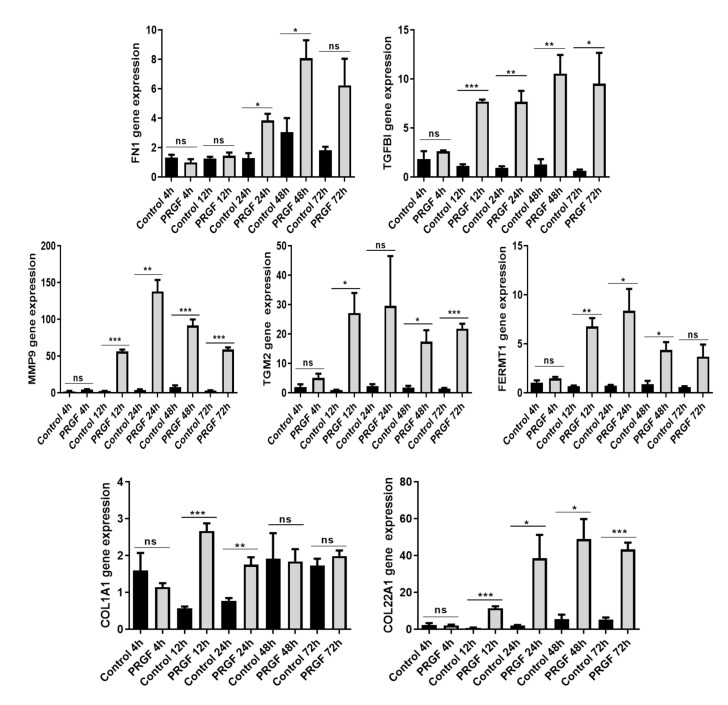
Time kinetics of PRGF-induced ECM-related proteins in human keratinocytes. Human primary keratinocytes were stimulated with PRGF for the indicated periods. Relative gene expression was analyzed by real-time PCR. Shown are means ± s.e.m of three stimulations (* *p* < 0.05, ** *p* < 0.01, *** *p* < 0.001; ns = non-significant; Student’s *t*-test).

A time kinetic study from 4 to 72 h revealed a significant PRGF-induced gene expression of almost all investigated genes after 2–3 days incubation time (Figure 3). Except for FN1, all genes showed a significant induction already after 12 h. Notably, no gene was significantly induced by PRGF already after 4 h (Figure 3).

### 2.4. Blockade of the Epidermal Growth Factor Receptor (EGFR) Influences PRGF-Mediated Induction of ECM-Related Factors in Keratinocytes

**Figure 4:**
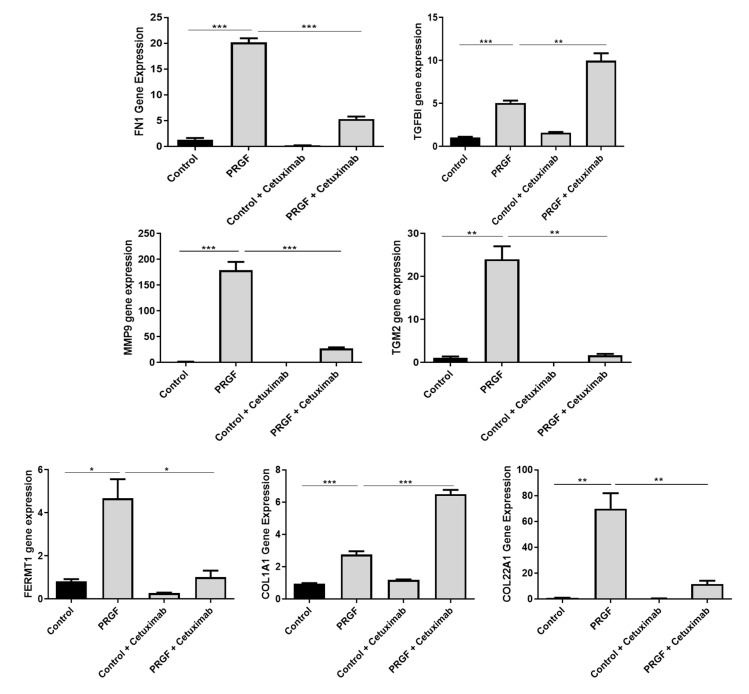
The epidermal growth factor receptor (EGFR) differentially influences the PRGF-induced expression of ECM-related proteins in human keratinocytes. Human primary keratinocytes were stimulated for 20 h with PRGF in the presence or absence of the EGFR blocking antibody cetuximab. Relative gene expression was analyzed by real-time PCR. Shown are means ±s.e.m of three stimulations (* *p* < 0.05, ** *p* < 0.01, *** *p* < 0.001; Student’s *t*-test).

In previous studies we observed a major influence of the epidermal growth factor receptor (EGFR) on the PRGF-mediated induction of antimicrobial peptides in keratinocytes [22,23,24]. Therefore we hypothesized that the PRGF-mediated expression of ECM-related factors is also influenced by EGFR signaling. Blockade of the EGFR by the monoclonal EGFR-antibody cetuximab led to significant inhibition of the PRGF-mediated induction of FN1, MMP9, TGM2, FERMT1 and COL22A1 in human primary keratinocytes. In contrast, the induction of TGFBI and COL1A1 by PRGF was further enhanced when the EGFR was blocked by cetuximab (Figure 4).

### 2.5. Increased Expression of ECM-Related Genes In Ex Vivo Skin Explants

**Figure 5:**
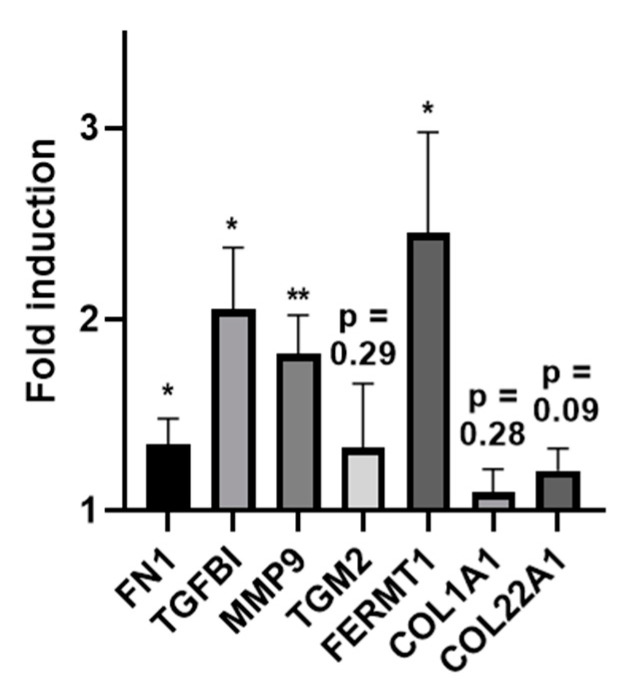
Induction of ECM-related proteins in human skin explants treated with PRGF. Human skin explants derived from surgery were treated with PRGF for approx. 20 h. Gene expression was analyzed by real-time PCR and results are shown as fold-induction compared to the unstimulated control. Shown are the means of 4 independent experiments with skin explants derived from 4 different donors (* *p* < 0.05, ** *p* < 0.01; Student’s *t*-test).

We then used skin explants derived from surgery to investigate the influence of PRGF in an ex vivo setting. Incubation of skin explants with PRGF preparations significantly induced FN1, TGFBI, MMP9 and FERMT1 gene expression with FERMT1 showing the highest induction. Expression of TGM2, COL22A1 and COL1A1 showed a low, non-significant increase (Figure 5).

### 2.6. Increased Expression of ECM-Related Genes in Experimentally Generated Skin Wounds In Vivo and after Vivostat PRF^®^ Treatment

**Figure 6:**
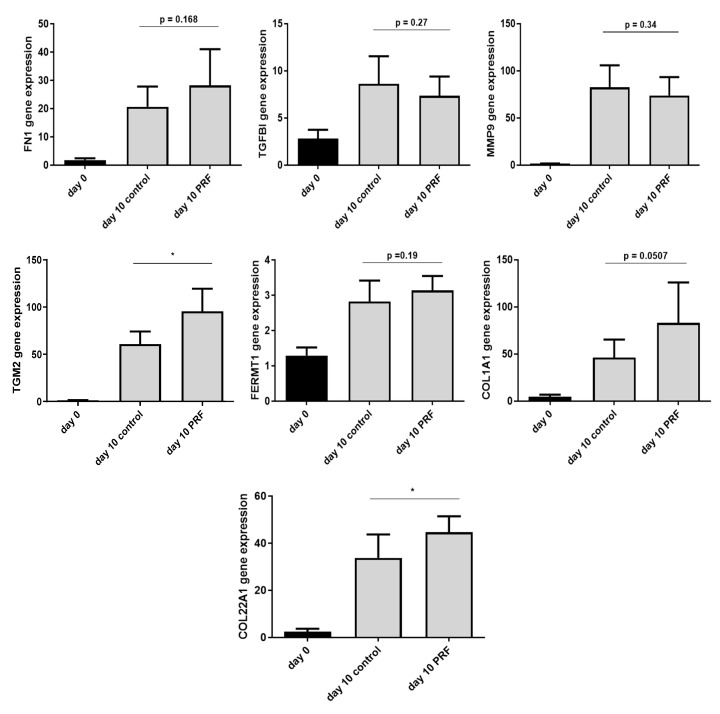
In vivo influence of Platelet-Rich Fibrin (PRF) on experimentally generated wounds. Experimentally generated bilateral gluteal wounds (biopsy punch, Ø 4 mm) of 10 test persons were treated by day 0 and day 5 with Vivostat PRF^®^ or NaCl 0.9% as control. After 10 days, wound areas were resected (biopsy punch, Ø 6 mm) and gene expression of the indicated factors was determined by real-time PCR (* *p* < 0.05, Student’s *t*-test).

Finally, we sought to determine the expression of the ECM-related factors in vivo. To this end, wounds were generated experimentally and gene expression analyzed after 10 days in either untreated wounds (controls) or Vivostat PRF^®^-treated wounds. All investigated factors showed an increased gene expression after 10 days of wounding. Expression of FN1, TGM2, FERMT1, COL1A1 and COL22A1 was further enhanced in Vivostat PRF^®^-treated wounds. However, the Vivostat PRF^®^-mediated increase of gene expression was only significant for TGM2 and COL22A1 (Figure 6).

## 3. Discussion

Stimulation of human primary keratinocytes with PRGF followed by whole transcriptome sequencing and analysis of gene ontology clustering revealed the upregulation of several factors involved in extracellular matrix (ECM) physiology, cellular interactions and angiogenesis. One might speculate that the induction of these ECM-related factors may contribute to the observed beneficial effects of autologous thrombocyte concentrate formulations (e.g., Vivostat PRF^®^) to treat chronic and hard-to-heal wounds [12,13,27,28]. Based on abundance and fold-upregulation we selected seven ECM-related factors for detailed expression analyses. These factors, transforming growth factor beta-induced protein (TGFBI), fibronectin 1 (FN1), matrix metalloproteinase-9 (MMP9), transglutaminase 2 (TGM2), fermitin family member 1 (FERMT1), collagen type I alpha 1 chain (COL1A1) and collagen type XXII alpha 1 chain (COL22A1) are all associated with ECM physiology and will be discussed in the following.

### 3.1. TGFBI

Transforming growth factor beta-induced protein (TGFBI, also known as βig-H3 and keratoepithelin) is an extracellular matrix molecule secreted by many cell types including keratinocytes [29,30,31] that regulates keratinocyte function [30], plays a critical role in extracellular matrix interactions [32], could increase adhesion, migration and proliferation of epithelial cells [33], could impair the function of fibroblasts in chronic wounds [34] and is therefore supposed to play an essential role in skin wound healing [34,35,36]. According to the whole transcriptome analyses gene expression of TGFBI was approx. 30-fold induced in PRGF-treated keratinocytes and showed the highest abundance of the 50 most upregulated genes. Real-time PCR analyses confirmed induction of TGBI gene expression in primary keratinocytes treated with PRGF. Although the exact role of TGFBI in keratinocytes and skin wounding remains to be determined, it is likely that the observed PRGF-mediated TGFBI induction in keratinocytes contribute to the beneficial effects of the clinical use of PRF in wound treatment.

### 3.2. FN1

Fibronectin 1 (FN1) is an extracellular matrix molecule that functions as a “master organizer” in matrix assembly since it forms a bridge between cell surface receptors as integrins or collagens and other focal adhesion molecules [37]. Together these protein networks form the ECM. One main function of fibronectin is to serve as a scaffold in ECM physiology thus mediating cell adhesion and migration [38,39]. FN1 is produced by various cell types including fibroblasts and keratinocytes and is involved in the wound healing process and angiogenesis by promoting opsonization of tissue debris as well as migration, proliferation and contraction of involved cells [38,40]. Therefore FN1 is indispensable for a successful wound healing process [41,42,43,44,45]. The whole transcriptome analysis revealed fibronectin 1 (FN1) as the third most abundant gene in the PRGF-treated keratinocytes suggesting a major role of fibronectin in keratinocytes biology.

### 3.3. MMP9

The whole transcriptome analysis of PRGF-stimulated keratinocytes revealed matrix metalloproteinase 9 (MMP9) as the seventh most induced protein by PRGF. MMP9 is a protease involved in many physiological processes including remodeling of the ECM. This is mainly achieved by the degradation of ECM proteins such as gelatin, collagen and elastin [46]. MMP9 plays also an important role by removal of the fibrinogen matrix [47]. MMP9 is involved in keratinocyte migration and tissue remodeling [48] and displays a key tissue remodeling enzyme that is critical for wound healing [49]. Many cell types are able to produce and secrete MMP9, including keratinocytes and fibroblasts.

### 3.4. TGM2

Transglutaminase-2 (TGM2 or TG2, also named tissue glutaminase) is a multifunctional protein [50] that has cross-linking and hydrolysis activity, is enrolled in many biological processes in the human body [51,52] and is implicated in the complex wound healing process [52,53,54,55]. TGM2 enhanced the proteolytic resistance and strength of the collagen matrix [56] and promotes angiogenesis and wound healing [51,57]. TGM2 is also expressed by keratinocytes and it is important to differentiate TGM2 from keratinocytes transglutaminase TGM1 which is a transglutaminase mainly expressed by keratinocytes and involved in the formation of the epidermal cornified cell envelope [58]. TGM2 is a protein cross-linking enzyme and is involved in ECM stabilization by mediating the interaction of integrins with fibronectin [59]. Interestingly, TGM2 suppression greatly reduced MMP9 expression in a lung cancer cell line [60] suggesting that TGM2 may influence MMP9 expression also in skin. The whole transcriptome sequencing identified TRM2 as one of the 25 most induced genes in PRGF-stimulated human primary keratinocytes. Real-time PCR analyses confirmed this PRGF-mediated induction of TGM2 in keratinocytes.

### 3.5. FERMT1

FERMT1 (fermitin family member 1 or kindlin-1) is a focal adhesion protein mainly expressed in basal keratinocytes with accumulation at cell-matrix adhesion sites that increases the integrin activity causing cell adhesion, spreading and migration [61,62]. In the context of wound healing it regulates the assembly of the extracellular matrix (ECM) as well as survival, proliferation and differentiation of involved cells [63]. A deficiency or defect of FERMT1 is associated with Kindler syndrome, a skin disease characterized by cutaneous blistering, atrophied skin, photosensitivity, progressive poikiloderma in sun-exposed areas [62] and lethal neonatal intestinal epithelial dysfunction [64]. Moreover, it has been shown that Kindlin-1 is essential in EGF-induced re-epithelialization in skin wound healing [65] and regulates cutaneous stem cell proliferation [63] that is also supposed to be important for wound healing.

### 3.6. Collagens COL1A1 and COL22A1

Collagens are the most abundant proteins in the ECM. Collagen type I alpha 1 chain (COL1A1) is involved in the formation of type I collagen fibers [66,67] and is therefore involved in the profibrotic gene induction program [68]. Although mainly expressed by fibroblasts COL1A1 is also expressed in keratinocytes as confirmed by our study. Collagen type XXII alpha 1 chain (COL22A1) is also a member of the ECM and has been reported to act as a cell adhesion ligand for skin keratinocytes and fibroblasts [69]. It is speculated that COL22A1 is involved in the formation of myofibroblasts from fibroblasts and epithelial cells [70]. These cells have an important role in ECM matrix production and fibrosis [70]. Moreover, myofibroblasts can accelerate the wound healing process by contracting the edges of the wound [71]. Thus, COL22A1 may play at least an indirect role in supporting wound healing. As shown here, the expression of both, COL1A1 and COL22A1, are induced in human keratinocytes by PRGF and Vivostat PRF^®^.

Our PCR analyses confirmed induction of all investigated ECM-related factors in human primary keratinocytes treated with PRGF in vitro. A time kinetic analysis revealed highest PRGF-induced expression of the ECM-related factors after 12–48 h. After 4 h PRGF treatment of the keratinocytes, none of the investigated genes was significantly upregulated. This is in line with the PRGF-mediated induction of the antimicrobial peptides hBD-2 and hBD-3 in keratinocytes, which showed no induction after 4 h and 12 h [22,23]. Interestingly, IL-6 was strongly induced in keratinocytes by PRGF already after 4 h and the PRGF-mediated hBD-2 induction was–at least in part–dependent on activation of the IL-6 pathway [22]. To evaluate whether IL-6 signaling is also involved in the observed PRGF-induced expression of the ECM-related factors, we incubated the keratinocytes with the IL-6-receptor blocking antibody tocilizumab. In contrast to hBD-2, none of the PRGF-induced ECM-related genes was significantly influenced by tocilizumab (data no shown) suggesting that IL-6 signaling is not involved.

It is known that PRGF preparations contain several growth factors. We could recently demonstrate that the PRGF-mediated induction of various antimicrobial peptides was depended on the activation of the epidermal growth factor receptor [22,24]. Thus, we speculated that the EGFR may be involved in the observed PRGF-mediated induction of analyzed ECM-related factors. Indeed, our data revealed a significant influence of the EGFR signaling on the PRGF-induced ECM-related factors. Blockade of the EGFR by the monoclonal antibody cetuximab revealed a significantly decreased PRGF-mediated induction of FN1, MMP9, TGM2, FERMT1 and COL22A1. The EGFR-dependent MMP9 induction by PRGF is in concordance with a previous study reporting that the induction of MMP-9 by TNF-alpha in keratinocytes was dependent on the activity of the EGFR [72]. Similarly, EGFR activation was reported to activate TGM2 expression [73]. Our study also revealed that the induction of FERMT1 by PRGF required the EGFR. Interestingly, a recent study reported that FERMT1 itself had a positive influence on EGFR level in keratinocytes by direct interaction with the EGFR which protected EGFR from lysosomal degradation [62]. Thus, it seems that FERMT1 and EGFR interact with each other via a direct positive feedback loop. In contrast to FN1, MMP9, TGM2, FERMT1 and COL22A1, our results revealed that the PRGF-mediated induction of TGFBI and COL1A1 was significantly enhanced by blocking the EGFR suggesting that EGFR activation inhibited the PRGF-induced expression of TGFBI and COL1A1. The observed differential effects of EGFR blockade on the PRGF-induced expression of the ECM-related genes are in line with a previous study reporting differential effects of EGFR inhibition on the skin barrier and skin inflammation. On the one hand blockade of EGFR signaling resulted in the down-regulation of antimicrobial peptides and tight junction genes, on the other hand skin-associated chemokines were induced thus explaining cutaneous inflammation after anti-EGFR therapy [74]. Together, our data indicate that the influence of PRGF on the expression of ECM-related factors strongly depends on the EGFR. This is in line with the known importance of EGFR signaling in the context of the ECM [75,76]. It remains to be shown whether EGFR-ligands present in the PRGF directly activate the EGFR or if a PRGF-mediated release of EGFR ligands influence expression of the ECM-related factors in an auto-/paracrine manner. Either way, our data supports the hypothesis that the PRGF-mediated cutaneous EGFR activation is associated with the beneficial effects associated with PRGF/PRF-related therapies in the context of wound healing.

Our study also revealed a positive influence of PRGF and PRF in an ex vivo setting using skin explants and in an in vivo setting with experimentally generated wounds. Interestingly, expression of TGM2, COL1A1, COL1A22 were not induced in the ex vivo experiments but significantly (in the case of COL1A1 with *p* = 0.0507 almost significantly) induced in the in vivo situation. A possible explanation for these observed differences may be related to the fact that the influence of PRGF on the ECM-related factors was investigated in the ex vivo setting after approximately one day incubation time. In contrast, the expression of the ECM-related factors in the in vivo setting was analyzed only 5 days after the last PRF treatment. Another explanation for the different results may be related to potential differences in the composition of PRGF and Vivostat PRF^®^. However, both formulations are based on concentrated platelets. Therefore, we expect that the main effector molecules are present in both formulations. In this regard it would be interesting to perform a detailed analysis of the major active factors present in both formulations.

Taken together, our study identifies the ECM organization as a major target of the effects elicited by PRGF and Vivostat PRF^®^ treatment. ECM remodeling and an intact ECM is important for restoration of the skin barrier after wounding. Thus, our data highlight the PRGF-mediated induction of ECM-related factors as underlying effect that may contribute to the beneficial effects of thrombocytes-derived factors in wound healing. Clearly, future studies are needed to further investigate the influence of thrombocytes lysates on the wound healing process and to decipher the underlying mechanisms.

## 4. Materials and Methods

### 4.1. Preparation of PRGF

The preparation of the PRGF used for the in vitro experiments was prepared as described before [22]. Briefly, PRGF was generated from freshly isolated human thrombocyte concentrates by centrifugation and ultrasound treatment under sterile conditions followed by repeated freezing and thawing.

### 4.2. Culture and Stimulation of Primary Human Keratinocytes

Human primary keratinocytes derived from foreskin and pooled from several donors were obtained from Promocell (Heidelberg, Germany). Cells were cultured in Keratinocyte Growth Medium 2 (KGM-2, Promocell) at 37 °C with 5% CO_2_ and stimulated with the indicated dilutions of PRGF in 12-well tissue culture plates (BD Biosciences, Franklin Lakes, NJ, USA) at 90–100% confluence. Subsequently, total RNA was isolated and reverse transcribed in cDNA as described [22]. In order to analyze the participation of the epidermal growth factor receptor (EGFR) and the IL-6 pathway, the EGFR-blocking antibody cetuximab (Merck, Darmstadt, Germany) or the IL-6 receptor blocking antibody tocilizumab (Hoffmann-La Roche, Basel, Switzerland) were used at a concentration of 20 µg/mL and 50 µg/mL, respectively.

### 4.3. Whole Transcriptome Sequencing (RNA-Seq)

Total RNA isolation for RNA-Seq was done with the NucleoSpin RNA isolation kit according to the manufacturers’ protocol. RNA libraries were prepared using the Illumina Truseq^®^ Stranded mRNA protocol including poly-A enrichment. All 10 libraries were pooled and sequenced on one lane on a HiSeq4000 producing 1 × 50 bases single-reads according to the manufacturer’s protocol (Illumina, San Diego, CA). Raw mRNA sequencing data were processed as follows—Illumina standard adapters were trimmed using Cutadapt (version 1.15). Reads were mapped to the human reference genome (GRCh38, Ensembl release 91) using Tophat2 [77] (version 2.1.1) and Bowtie 2 [78] (version 2.3.2). Mapped reads were cleaned and sorted using Samtools [79] (version 1.5). Number of reads for each gene was counted using HTSeq [80] (version 0.10.0) and annotated according to the Gencode version 27 annotation gtf file.

Outliers were assessed using principal component analysis (PCA). Differential expression analysis of stimulated vs. unstimulated keratinocytes was conducted using the DESeq2 [81] Bioconductor package (version 1.24.0). Analysis was performed using the parametric Wald test, independent filtering of the results and a log2 fold change threshold of 0.5 (lfcThreshold = 0.5, altHypothesis = “greaterAbs”). A false discovery rate (FDR) < 5% was used to declare significance. Log fold change estimates were corrected using the DESeq2 inbuilt log2 fold change shrinkage function with the *apeglm* [82] method. Gene enrichment analysis was performed using the Clusterprofiler [83] Bioconductor package (version 3.12.0) for biological processes compiled from Gene Ontology [84].

### 4.4. Real-Time PCR

Gene expression was determined by real-time PCR using a fluorescence-temperature cycler (StepOne Plus, Life Technologies) as described previously [85]. The intron spanning primers used are presented in Table 1. An annealing temperature of 60 °C was used for all reactions.

Serial dilutions of cDNA were used to generate standard curves for relative quantification. Expression levels of the specific genes were adjusted to the house-keeping gene RPL38 (ribosomal protein L38), which was amplified with the primer pair—5′-TCA AGG ACT TCC TGC TCA CA -3′ (forward primer) and 5′-AAverwA GGT ATC TGC TGC ATC GAA -3′ (reverse primer).

### 4.5. ELISA

The supernatants of PRGF-stimulated keratinocytes were analyzed by ELISA specific for fibronectin 1, (FN1), matrix metalloproteinase-9 (MMP9) and collagen type I alpha 1 (COL1A1) using DuoSet ELISA kits from R&D systems (Minneapolis, MN, USA) according to the manufacturers’ instructions.

#### 4.5.1. Expression Analysis of ECM-Related Genes In Ex Vivo Skin Explants

Ex vivo skin explants were obtained from abdomen or breast reduction surgeries and used for stimulation experiments. This approach was approved by the local ethics committee of the Medical Faculty, University of Kiel, Germany (D 414/09; D 442/16) in accordance with the Declaration of Helsinki Principles guidelines. Samples were washed with phosphate-buffered saline and subcutaneous fat tissue was removed. Subsequently, skin samples were cut in defined pieces (0.25 cm^2^). For stimulation, the skin samples were placed in reaction tubes filled with 240 µL KGM-2 without supplements together with 60 µL of PRGF and were incubated at 37 °C in a humidified atmosphere with 5% CO2 for 24 h. RNA Isolation was performed with NucleoSpin RNA Kit (Macherey-Nagel, Düren, Germany) according to the manufacturers’ protocol. cDNA analysis was performed as described above.

#### 4.5.2. Expression Analysis of ECM-Related Genes in Experimentally Generated Skin Wounds and after PRF Treatment In Vivo

Experimentally wounds were generated and treated with Vivostat PRF^®^ as described before [22]. Briefly, bilateral human gluteal wounds were generated by a 4 mm biopsy punch in 10 male human students. Right gluteal wounds were treated with sterile NaCl as control whereas left gluteal wounds were treated with Vivostat PRF^®^. Vivostat PRF^®^ and NaCl treatment was repeated on day 5. On day 10 we resected bilateral gluteal wounds using a 6 mm biopsy punch for keratinocyte isolation followed by RNA-isolation, reverse transcription, cDNA synthesis and gene analyses. These in vivo experiments were positively evaluated and approved by the local Ethics Committee of the Medical Faculty (A 115/13) in accordance with the Helsinki guidelines. Written informed consent was obtained from the study participants.

## Figures and Tables

**Table 1:** Primer sequences used to analyze gene expression of the indicated ECM-related factors by real-time PCR.

Gene	Forward Primer	Reverse Primer
Transforming Growth Factor Beta Induced, TGFBI	ACCCAGAAGCCCTGAGAG	TGCAGCCCACCTCCAGTG
Fibronectin 1, FN1	ACAACGTCATAGTGGAGGCA	CATCCGTAGGTTGGTTCAAG
Matrix Metalloproteinase 9, MMP9	GACACGCACGACGTCTTCCA	CACTGCAGGATGTCATAGGTCA
Transglutaminase 2, TGM2	CTCAACCTGGAGCCTTTCTC	AGGGCCCGCACCTTGATGA
Fermitin Family Member 1, FERMT1	GATTCCAGTGACAACATGGAG	TCAAACTCGATGACCACCTG
Collagen Type I Alpha 1 Chain, COL1A1	CTGGAAGAGTGGAGAGTACTG	GTCTCCATGTTGCAGAAGAC
Collagen Type XXII Alpha 1 Chain, COL22A1	CAGGAGAGAAAGGAGTCCC	TGCCCCAGGCTGGCCTTTTC

## Supplementary Materials

Supplementary materials can be found at https://www.mdpi.com/1422-0067/21/12/4404/s1, Table S1: Shown is a list of all significant influenced genes in PRGF-treated human primary keratinocytes identified by RNA-seq.
